# Assessment of the Atmospheric Deposition of Potentially Toxic Elements Using Moss *Pleurozium schreberi* in an Urban Area: The Perm (Perm Region, Russia) Case Study

**DOI:** 10.3390/plants13172353

**Published:** 2024-08-23

**Authors:** Evgeniya Gatina, Inga Zinicovscaia, Nikita Yushin, Omari Chaligava, Marina Frontasyeva, Alina Sharipova

**Affiliations:** 1 Department of Biogeocenology and Nature Conservation, Perm State University, 15, Bukireva St., 614068 Perm, Russia; suslovael@mail.ru (E.G.); sharipova@ail.ru (A.S.); 2 Joint Institute for Nuclear Research, Joliot-Curie 6, 141980 Dubna, Russiachaligava@jinr.ru (O.C.); marina@nf.jinr.ru (M.F.); 3 Horia Hulubei National Institute for RD in Physics and Nuclear Engineering, 30, Reactorului Str., 077125 Magurele, Romania; 4 Faculty of Informatics and Control Systems, Georgian Technical University, 77, Merab Kostava Str., 0171 Tbilisi, Georgia

**Keywords:** moss biomonitoring, air pollution, ICP-OES, contamination indices, ecological risk

## Abstract

Assessment of air quality in urban areas is very important because pollutants affect both the environment and human health. In Perm (Russia), a moss biomonitoring method was used to assess the level of air pollution. The concentrations of 15 elements in 87 samples of moss *Pleurozium schreberi* in the city territory were determined using a direct mercury analyzer and an inductively coupled plasma atomic emission spectroscopy. Using factor and correlation analyses, the grouping of elements and their relationship with emission sources were established. The main sources of emissions of potentially toxic elements are the transportation (road and rail), metallurgical, and chemical industries. The level of atmospheric air pollution was assessed by calculating the environmental risk index, pollutant load index, and pollution coefficient. Based on the values of the pollution index, the level of atmospheric air pollution in Perm varies from unpolluted to highly polluted, with moderate environmental risk.

## 1. Introduction

Air pollution is directly linked to urbanization and is one of the most important environmental problems [1]. According to the World Health Organization (WHO) [2], air pollution results in 4.2 million premature deaths worldwide, and 89% of them are in low- and middle-income countries. According to WHO estimations, the deaths are mainly caused by chronic obstructive pulmonary disease, ischemic heart disease, cancer, and acute respiratory infections.

Potentially toxic elements (PTEs) are among the main air pollutants and may originate in the environment from a mixture of natural and anthropic emission sources, including forest fires, volcano eruptions, soil erosion, vehicles, industry, fuel combustion, mining, and agricultural activities [3,4,5,6]. The hazards of PTEs are attributed to their persistence in the environment and bioaccumulation in food chains, which can have negative impacts on ecosystems, food quality, and human health [7].

Numerous initiatives have been taken to combat air pollution, but at the same time, pollution levels continue to be a problem in many cities. Static monitoring stations currently utilize equipment to monitor a suite of air pollutants, including CO, NOx, NO and NO_2_, O_3_, PM10, and PM2.5 [8]. However, these stations are often very expensive (the price ranges from EUR 5000 to EUR 30,000) and require regularly maintenance. Listed limitations as well as specific criteria for their installations results in the operation of air monitoring stations in more densely populated urban and residential areas, which satisfies legal requirements but allows accurate data only for a limited number of locations, leading to a lack information about localized gradients that may be important for health protection. Moreover, many cities in developing countries do not have such stations due to financial reasons [8,9].

Biomonitoring is increasingly recognized as a cost-effective alternative method that can facilitate large-scale and long-term quantitative and qualitative assessments; therefore, this method can help overcome the financial and instrumental limitations of conventional monitoring [10].

Among plants, mosses are ecological indicators widely used to assess air pollution in urban areas [11,12]. Because of their distinct physiological characteristics, such as a lack of a well-developed root system, which contributes to nutrition uptake from wet and/or dry depositions, mosses are a perfect natural indicator of the quality of the air [13,14]. Other advantages of mosses include their ubiquity, ability to transport and accumulate PTEs at very high concentrations, high cation exchange capacity, and a large surface-to-volume ratio [15,16,17]. In addition, due to their slow growth rate, moss growth segments can be used as an estimate of the integrated exposure to toxic metals over longer periods, and not just the current state at the time of collection, which is particularly important in areas where levels of emissions of potentially toxic elements change rapidly [13,16].

Two types of biomonitoring that use moss as a bioindicator to evaluate atmospheric contamination are differentiated in the literature: (i) passive biomonitoring, which refers to the use of moss that grows naturally in a particular area, and (ii) active biomonitoring, which refers to the transplantation of moss from other locations to areas of interest. Passive biomonitoring is more appropriate for extensive studies in large areas (i.e., regional or national studies), while active biomonitoring is more appropriate for studies in smaller areas (i.e., urban or industrial areas) [18,19].

The majority of the research on passive biomonitoring is performed within the framework of the United Nations Economic Commission for Europe International Cooperative Program on Effects of Air Pollution on Natural Vegetation and Crops (UNECE ICP Vegetation), which was established in 1990 and covers most European countries. Twenty-one European countries contributed to the first campaign of the project [20]. Since 1990, moss surveys have been performed every five years, and the number of countries involved in the program continuously increases [14]. In addition to their use in monitoring on the national level, mosses have been extensively used for environmental monitoring in many urban areas [12,13,21,22,23]. However, active monitoring is mainly performed in urban areas due to the scarcity or absence of moss [19].

The aim of the present study was to assess the level of air pollution in Perm, the industrial center of the Urals (Russia), using the passive moss biomonitoring technique. To achieve this goal, the following specific objectives were pursued: (1) a determination of the concentrations of PTEs in mosses collected in Perm using several analytical techniques; (2) the identification of possible sources of PTE emissions using statistical tools; and (3) an assessment of the level of air pollution and the associated human health risks.

## 2. Results and Discussion

### 2.1. Basic Statistics

Table 1 shows the minimum, maximum, median, and mean values for elements identified in moss samples as well as the standard deviation (SD), coefficient of variation (CV), and background values (in mg/kg). The results obtained for moss samples collected in the Gainsky district (a relatively clean region of the Perm region) were used as background data.

The mean concentrations of elements in the moss samples were ranked in the following order: P > Al > Fe > S > Mn > Ba > Zn > Sr > Cu > V > Pb > Cr > Co > Cd > Hg. Phosphorous is an important nutrient for moss development, and it was shown that *P. schreberi* has a higher capacity to absorb P than other moss species [24,25]. Other elements determined in moss samples can have both natural and anthropic origins. The concentrations of elements in moss samples collected in the background area were consistently lower than the mean values for the majority of the elements; Co (81%), Fe (79%), Cr, V (75% for both elements), Al (65%), and Mn (62%) had the greatest differences. P (9.1%) had the smallest difference.

For all the tested elements, the CV values were below 75% (from 21% to 83%), indicating moderate variability in the data set. It is considered that a moderate variation reflects similar contamination levels for all elements throughout the study area [26]. High CV values for Mn (83%) and Al (75%) suggest an effect due to a polluted air mass derived from long transportation durations or regional air pollution [27].

### 2.2. Correlation and Factor Analyses

**Figure 1:**
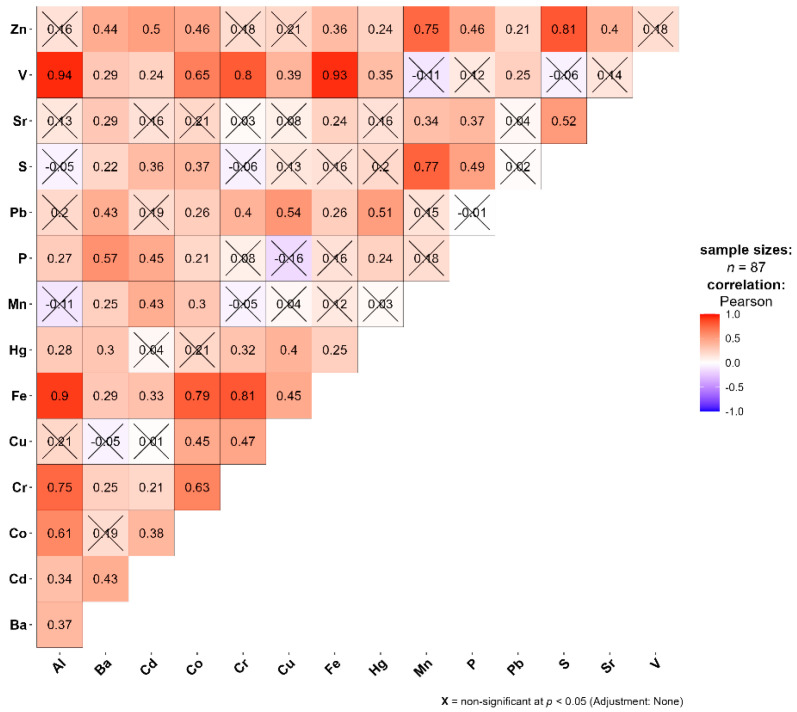
Correlation analysis for moss samples collected in Perm.

Across the sampling region, there were a number of significant (*p* < 0.05) and strong (r ≥ 0.7) correlations between the tested elements (Figure 1).

**Figure 2:**
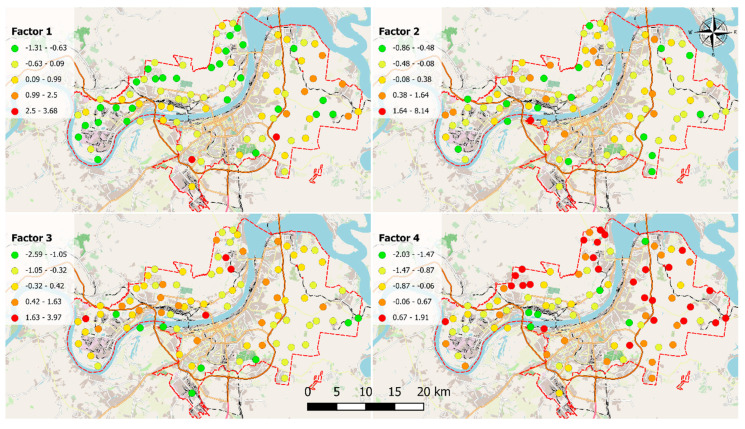
The distribution of the factor scores for Factors 1–4.

Strong positive correlations (r ≥ 0.7) were evident between Al and V, Al and Fe, and Al and Cr. There were also strong and significant correlations between Co and Fe, Cr and Fe, Cr and V, Fe and V, and Fe and Cr. Additionally, Mn showed a high correlation with S and Zn (Figure 2). No correlations were evident between Ba, Cd, Hg, P, and Sr and any other metals.

The relationships between these elements were further investigated using factor analysis. Four main factors that represent 78% of the total variance were identified. The matrix of rotated factor loadings is presented in Table 2, and the distribution of factor scores is displayed in Figure 2.

The association of Al, Co, Cr, Fe, and V is represented by Factor 1 (Figure 2), which has 45% of the variance. The primary sources of these elements are industrial activity and road dust. According to Charron et al. [28], traffic is a significant source of PTEs in urban environments due to both exhaust and non-exhaust emissions. In urban areas, the elemental composition of road dust is very similar to that of soil [29]. Even though Al and Fe are considered crustal metals [3], in urban areas, Fe could originate from metallic brakes and steel fibers [28]. Chromium is a component of brake linings and also originates from oil combustion [28]. Furthermore, Cr is also a component of yellow road markings [30]. Vanadium is present in the residual and fuel oils of all types of engines [31]. The high concentrations of elements grouped in Factor 1 were determined in samples collected near highways and secondary roads.

Thus, vehicles are one of the most important sources of PTEs in Perm. High levels of elements were also found near railways and the Osentsovsky industrial hub (one of the largest in Russia), which includes 46 metallurgical, chemical, construction, and oil refinery companies. High levels of Fe, Co, and Cr were found near the railway in Poland [32]. Oil products are a significant source of vanadium in the atmosphere [31], while construction materials can contribute to Al, Fe, Co, and Cr emissions [33].

Twenty-two percent of the variance can be attributed to Factor 2, which is associated with high loadings of Mn, S, and Zn. The highest concentrations of elements were determined near the Perm Chemical Company, Perm powder mill, and gravel plant. Sulfur is one of the main components used for black powder production [34]. Zinc compounds are mainly used for galvanization (50%), automotive tire production (20%), die casting (17%), and brass production (17%), as well as the production of tiles, ceramics, and glass [35]. Manganese is widely used in the production of non-ferrous metallurgy, steel, batteries, electrode materials, catalysts, Mn alloy production, welding, coke ovens, and Mn salt production [36,37]. In addition to industrial processes, both metals can be emitted by vehicles. Zinc is the most abundant PTE emitted from tires due to the addition of ZnO and ZnS during vulcanization [4]. Methylcyclopentadienyl manganese tricarbonyl is used as an additive in unleaded gasoline [38]. Thus, the chemical and defense industries, as well as vehicles, are important emitters of elements included in Factor 2.

Copper, Hg, and Pb have high loadings in Factor 3, which accounts for 12% of the total variance. The highest concentrations of elements were determined near Promtec (production of compressors and gas pumping equipment), Kamskaya Chemical Company (production of chemicals, paints, laboratory equipment and appliances, and household chemicals), transport maintenance facilities, Uralmechanics (metal processing and coating), and Perm Plant “Zvezda” (production of metal structures, products, and parts for buildings). Fuel combustion and non-ferrous metal production are the major anthropogenic sources of Cu emissions found in the atmosphere [39]. Lead is widely used in the chemical industry, the manufacturing of batteries and paints, the processing of metals, and the production of leaded aviation gasoline [40,41]. The four main anthropogenic sources of Pb in the environment are the combustion of leaded gasoline, coal combustion, metallurgical activities, and waste incineration [5]. Anthropogenic Hg emissions can be associated with non-ferrous metal smelting, the iron and steel industries, the chlor-alkali industry, and fossil fuel burning [42,43].

Factor 4 (which accounts for 13% of the explained variance) is represented by Ba and P. Barium is a component of the filler of brake linings [28]. Mineral dust and coal combustion are the dominant sources of atmospheric P emissions [44]. Since high concentrations of these elements were found near highways, road and mineral dust can be considered the main sources of their emissions in Perm.

### 2.3. Assessment of the Level of Air Pollution

To assess the level of air pollution in Perm, the CF and PLI were calculated (Table 3). CF values for Hg and P indicate the absence or suspicion of pollution. For Cd and Sr, the contamination ranged from no contamination to slight contamination, while for Ba, Cu, Pb, S, and Zn, the CFs indicated low to moderate contamination. A severe level of pollution was found for Al, Co, Cr, Fe, Mn, and V. The highest CF values were obtained for moss samples collected in the vicinity of highways (Fe, Al, Cd, and Mn) and industrial enterprises (Co, Cr, Pb, S, Sr, Zn, and Hg). Based on the PLI values, Perm can be characterized as unpolluted and moderately polluted.

The PER values ranged from 3.6 to 16.3 for Cd, from 4.1 to 31 for Cr, from 33 to 112 for Cu, from 13.6 to 74 for Pb, and from 34 to 201 for Zn. RI values ranged from 100 to 316, with an average value of 170. Regarding the sampling sites, 28% were characterized by a low RI, 71% by a moderate RI, and 1% by a considerable RI. The highest RI was calculated for samples collected near an industrial cluster, indicating the negative impact of industrial activity on air quality and human health.

## 3. Materials and Methods

### 3.1. Study Area

Passive moss biomonitoring was performed in Perm, a city with an area of about 800 km^2^ located in the Cis-Ural region, which is one of the largest industrial centers of the Russian Federation and the Perm region. Perm is located on the border of two forest regions: the taiga zone, which is the southern taiga region of the European part of the Russian Federation, and the coniferous broadleaved forest zone, which is the region of coniferous–deciduous (mixed) forests of the European part of the Russian Federation (southern part). Urban forests make up slightly more than 330 km^2^ (more than 40% of the city’s territory). The climate in Perm is moderately continental, with an average temperature of +17 °C in July and −15 °C in January. Annual precipitation is 500–600 mm. Loamy and soddy-podzolic soils prevail.

Perm is one the largest industrial centers, where the leading industries are machine building, oil and gas refining, the chemical and petrochemical industry, electric power engineering, metalworking, woodworking, and the paper industry, as well as manufacturing industries [45]. The largest producers of industrial products are enterprises within the defense complex, as well as the Joint Stock Company (JSC) Novomet-Perm, JSC Perm Mashinostroitel Plant, SIBUR-Khimprom JSC, HaloPolymer Perm JSC, and Kamtex-Khimprom JSC [46,47].

### 3.2. Sampling and Chemical Analysis

**Figure 3:**
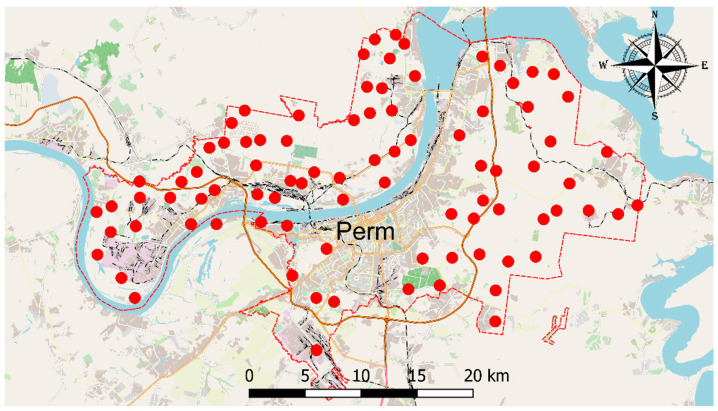
Moss sampling map of Perm, Russia.

*Pleurozium schreberi* (*P. schreberi*) (Brid.) Mitt. is representative of the family Hylocomiaceae, which comprises the red-stemmed feather moss. *P. schreberi* is the most common moss species and is predominant in the ground layer of boreal forests and subalpine and arctic ecosystems [48]. Two sampling campaigns were carried out during July of 2022 and 2023. *Pleurozium schreberi* moss samples were collected at 87 sites, mainly located in urban forests (Figure 3). Moss sampling was performed according to a manual developed by the ICP Vegetation program [14]. For the analysis, green and green-brown moss segments corresponding to a three-year growth period were selected. The moss was thoroughly cleaned of foreign debris and soil residues. Moss samples from the Gainsky district (a relatively clean area of Perm region) in the northwestern part of Perm Krai were taken as background data.

The elemental composition of the moss samples was determined using inductively coupled plasma optical emission spectrometry (ICP-OES) and a DMA-80 Milestone direct mercury analyzer. Mercury content in moss samples was determined according to a procedure described in [23]. For ICP-OES analysis, 0.5 g of moss was placed in a Teflon vessel with 5 mL of HNO_3_ and 2 mL of H_2_O_2_. The samples were digested in a MARS-6 microwave system (CEM, Matthews, Pittsburgh, PA, USA). After digestion, the resulting solutions were filtered through 0.45 µm filter paper and diluted to a final volume of 50 mL with bi-distilled water. The concentrations of 14 elements, i.e., Al, Ba, Cd, Co, Cr, Cu, Fe, Mn, P, Pb, S, Sr, V, and Zn, were determined via ICP-OES using a Plasmaquant PQ 9000 Elite (Analytik Jena, Jena, Germany). To ensure the quality control of the results, the certified reference material INCT-PVTL-6 (Polish Virginia Tobacco Leaves) was analyzed (Table 4). The recovery of the elements ranged from 85 to 116%.

### 3.3. Statistical Analysis

Processing of the data was performed using Excel (version 17) (Microsoft, Redmond, WA, USA) and IBM SPSS software (version 25) (IBM, Armonk, NY, USA). Descriptive statistics for the elements in the samples from 87 locations were calculated (Table 1). To discover associations between the chemical elements and to decrease the number of variables for the resulting data, factor analysis and correlation analysis were used. Multivariate statistical methods are applied in environmental studies to simplify large data sets with the purpose of identifying pollution sources and their relative elemental composition and to determine the contribution of each source to the total pollution level [49]. Since many statistical techniques, including factor analysis, are sensitive to non-normally distributed data, the Box-Cox transformation was performed. The ArcGIS software (Esri, Redlands, CA, USA) was used to build maps showing the spatial distributions of elements using the radial basis functions method.

### 3.4. Data Evaluation

To assess the level of air pollution, the contamination factor (*CF*) and Pollution load index were calculated. According to [50], the *CF*, a single index, is the ratio of an element’s concentration in the sample to its background value and is given by

(1) $$CF=\frac{C_{m}}{C_{b}}$$

where *C_m_* is the concentration of a given element and *C_b_* is the background concentration for the same element.

The degrees of contamination fell into the following categories: *CF* < 1—no contamination; 1–2—suspected contamination; 2–3.5—slight contamination; 3.5–8—moderate contamination; 8–27—severe contamination; and >27—extreme contamination [50].

The pollution load index (PLI) was used to assess the comprehensive level of PTEs for the studied area. The PLI represents the *n*^th^ order geometric mean of the entire set of CF [51] and is given by

(2) $$\mathrm{PLI}=\sqrt[{n}]{\prod_{i=1}^{n}C_{F,i}}$$

where *n* is the total number of elements.

The PLI data were divided into multiple categories: PLI < 1—unpolluted, 1 < PLI < 2—unpolluted to moderately polluted, 2 < PLI < 3—moderately polluted, 3 < PLI < 4—moderately to highly polluted, 4 < PLI < 5—highly polluted, and PLI < 5—very highly polluted [52].

The ecological risk of a particular element in moss is determined by the ecological risk index (RI), which takes into account the response of the environment and the toxicity of metals and is given by

(3) $$\mathrm{RI}=\sum {\mathrm{PER}}_{f}^{i}$$

(4) $${\mathrm{PER}}_{f}^{i}={\;C}_{f}^{i}\times {\;T}_{f}^{i}$$

where ${\mathrm{PER}}_{f}^{i}$ is the potential ecological risk index of each element, $C_{f}^{i}$ is the contamination factor, and $T_{f}^{i}$ is the “no toxic-response” coefficient for the given single metal. The toxic response factors are 2 for Cr, 5 for Cu, 30 for Cd, 1 for Zn, and 5 for Pb. There are four categories for ecological risk: RI < 150—low ecological risk; 150 ≤ RI < 300—moderate ecological risk; 300 ≤ RI < 600—considerable ecological risk; and RI ≥ 600—very high ecological risk [53].

## 4. Conclusions

Urban areas are exposed to serious environmental pollution due to intensive population growth and industrial development. The concentrations of 15 elements in moss samples taken at 87 sites in Perm were determined. The correlation and factor analysis results established the prevalence of anthropogenic sources of PTE emissions in Perm, which include the chemical, defense, and metallurgical industries, as well as automobile and railway transportation.

This is in agreement with national reports. Transportation is considered a primary source of Fe, Al, Cd, and Mn, and industrial enterprises are considered primary sources of Co, Cr, Pb, S, Sr, Zn, and Hg. The air pollution levels ranged from unpolluted to severely polluted; severe pollution was mainly found in industrial areas. These results are supported by the RI data, which indicate considerable ecological risk in industrial zones. The results show that city authorities should pay more attention to air pollution, which can cause serious health problems, especially among citizens living in close proximity to industrial enterprises.

## Figures and Tables

**Table 1:** Basic statistics, which serve as background values (in mg/kg), for moss samples collected in Perm.

Element	Min	Max	Mean	Median	SD	CV %	Background
Al	564	9038	2322	1803	1743	75	815.0
Ba	19.7	169.1	70.2	72.8	34.3	49	32.3
Cd	0.12	0.55	0.24	0.22	0.09	36	0.16
Co	0.34	3.75	1.28	1.12	0.68	53	0.25
Cr	2.1	15.6	5.6	5.3	2.6	47	1.43
Cu	6.6	22.6	11.4	10.9	3.1	27	5.18
Fe	490	6088	1975	1685	1196	61	410
Mn	51	3137	434	350	359	83	164
P	1405	4042	2700	2752	574	21	2455
Pb	2.7	14.7	5.8	5.7	1.9	34	3.35
S	1009	4641	1654	1601	415	25	1216
Sr	19.4	87.5	35.8	32.8	12.8	36	26.22
V	2.1	24.3	7.3	6.2	4.7	65	1.77
Zn	34.7	201.8	65.2	60.2	22.5	35	40.2
Hg	0.018	0.096	0.041	0.039	0.012	30	0.07

**Table 2:** Matrix of rotated factor loadings (varimax-normalized).

Element	Factor 1	Factor 2	Factor 3	Factor 4
Al	0.91	−0.08	0.05	0.31
Ba	0.16	0.18	0.33	0.79
Cd	0.29	0.45	−0.04	0.45
Co	0.77	0.43	0.11	−0.07
Cr	0.84	−0.04	0.31	0.02
Cu	0.38	0.18	0.65	−0.44
Fe	0.95	0.19	0.12	0.06
Hg	0.15	0.05	0.76	0.22
Mn	−0.07	0.89	0.05	0.02
P	0.08	0.34	−0.03	0.79
Pb	0.15	0.05	0.87	0.07
S	−0.04	0.93	0.04	0.12
Sr	0.09	0.52	0.04	0.26
V	0.94	−0.07	0.17	0.13
Zn	0.17	0.86	0.16	0.23
Prp. Total	0.29	0.22	0.14	0.13

**Table 3:** Contamination factor, pollution load index, and potential ecological risk index values for moss samples collected in Perm.

Element	CF			PER		
	Min	Max	Median	Min	Max	Median
Al	0.69	11.1	2.9			
Ba	0.61	5.2	2.2			
Cd	0.75	3.4	1.5	3.6	16.4	7.3
Co	1.4	15.0	5.1			
Cr	1.4	10.9	3.9	4.1	31.1	11.2
Cu	1.3	4.4	2.2	33	113	57
Fe	1.2	14.8	4.8			
Mn	0.31	19.1	2.6			
P	0.57	1.6	1.1			
Pb	0.81	4.4	1.7	13.6	73.6	29.0
S	0.83	3.8	1.4			
Sr	0.74	3.3	1.4			
V	1.2	13.7	4.1			
Zn	0.86	5.0	1.6	34.7	201.8	65.2
Hg	0.26	1.4	0.6			
PLI	1.05	3.3	2.0			

**Table 4:** Results of quality control for the INCT-PVTL-6.

	Determined Content, mg/kg	SD	Certified Value, mg/kg	Recovery, %
Al	213	1.6	252	85
Ba	46	0.2	41.6	111
Cd	2.41	0.017	2.23	108
Co	0.15	0.015	0.15	100
Cr	0.77	0.007	0.91	85
Cu	5.2	0.05	5.12	102
Fe	267	5.9	258	103
Mn	144	1.75	136	106
Ni	1.33	0.02	1.49	89
P	2798	21	2420	116
Pb	0.84	0.04	0.97	87
S	3886	88	3780	103
Sr	132	1.2	133	99
V	0.35	0.01	0.40	88
Zn	47.47	0.40	43.6	109

## Data Availability

The experimental data will be made available on reasonable request.
